# Evaluating drug distribution in children and young adults with diffuse midline glioma of the pons (DIPG) treated with convection-enhanced drug delivery

**DOI:** 10.3389/fnimg.2023.1062493

**Published:** 2023-02-28

**Authors:** Elwira Szychot, Dolin Bhagawati, Magdalena Joanna Sokolska, David Walker, Steven Gill, Harpreet Hyare

**Affiliations:** ^1^Department of Paediatric Oncology, Great Ormond Street Hospital for Children NHS Foundation Trust, London, United Kingdom; ^2^Department of Paediatric Oncology, Harley Street Children's Hospital, London, United Kingdom; ^3^Department of Paediatrics, Paediatric Oncology and Immunology, Pomeranian Medical University, Szczecin, Poland; ^4^Department of Neurosurgery, Charing Cross Hospital, Imperial College, London, United Kingdom; ^5^Department of Medical Physics and Biomedical Engineering, Faculty of Engineering Sciences, University College London, London, United Kingdom; ^6^Division of Child Health, School of Human Development, University of Nottingham, Nottingham, United Kingdom; ^7^Department of Translational Health Sciences, Institute of Clinical Neurosciences, Bristol Medical School, Faculty of Health Sciences, University of Bristol, Bristol, United Kingdom; ^8^Department of Neuroradiology, University College London Hospitals NHS Foundation Trust, London, United Kingdom

**Keywords:** convection-enhanced delivery, apparent diffusion coefficient (ADC), diffuse intrinsic pontine gliomas (DIPG), children, young adults

## Abstract

**Aims:**

To determine an imaging protocol that can be used to assess the distribution of infusate in children with DIPG treated with CED.

**Methods:**

13 children diagnosed with DIPG received between 3.8 and 5.7 ml of infusate, through two pairs of catheters to encompass tumor volume on day 1 of cycle one of treatment. Volumetric T2-weighted (T2W) and diffusion-weighted MRI imaging (DWI) were performed before and after day 1 of CED. Apparent diffusion coefficient (ADC) maps were calculated. The tumor volume pre and post CED was automatically segmented on T2W and ADC on the basis of signal intensity. The ADC maps pre and post infusion were aligned and subtracted to visualize the infusate distribution.

**Results:**

There was a significant increase (*p* < 0.001) in mean ADC and T2W signal intensity (SI) ratio and a significant (*p* < 0.001) increase in mean tumor volume defined by ADC and T2W SI post infusion (mean ADC volume pre: 19.8 ml, post: 24.4 ml; mean T2W volume pre: 19.4 ml, post: 23.4 ml). A significant correlation (*p* < 0.001) between infusate volume and difference in ADC/T2W SI defined tumor volume was observed (ADC, r = 0.76; T2W, r = 0.70). Finally, pixel-by-pixel subtraction of the ADC maps pre and post infusion demonstrated a volume of high signal intensity, presumed infusate distribution.

**Conclusions:**

ADC and T2W MRI are proposed as a combined parameter method for evaluation of CED infusate distribution in brainstem tumors in future clinical trials.

## Introduction

Convection-enhanced delivery (CED) of drugs to tumor tissue relies upon infusate bulk flow, to overcome the limitations of systemic delivery techniques. It permits the distribution of small and large molecular weight compounds to targeted areas of the central or peripheral nervous system, bypassing the blood brain barrier (BBB) (Lonser, 2017; Tosi and Souweidane, 2020). This novel technique enables local delivery of high concentration of the therapeutics while minimizing the systemic exposure, and hence, toxicity (Tosi and Souweidane, 2020; Szychot et al., 2021).

We have recently reported preliminary clinical experience of using CED infusions of carboplatin and sodium valproate for the treatment of children with diffuse midline glioma of the pons (Szychot et al., 2021). Although the safety and feasibility of this novel technique was demonstrated, quantifying the distribution of the infusate to determine the drug loading of tumor tissue is important to judge evidence for tumor response and symptomatology during and after infusions (Sampson et al., 2010; Iyer et al., 2011; van Putten et al., 2016; Jahangiri et al., 2017).

This technical requirement justified this retrospective study to develop evidence to support an imaging protocol for assessment of the distribution of infusate volume when diffuse midline gliomas of the pons (DIPG) are being treated with intermittent CED infusions as part of future clinical trials. The aim of this study was to determine whether T2W signal intensity (SI) and Apparent Diffusion Coefficient (ADC) could be used as a surrogate marker of infusate distribution.

## Patients and methods

This is a retrospective study of imaging data collected as part of a pilot study conducted under the Medicines and Healthcare products Regulatory Agency (MHRA) supervision with ethical approval of the Institutional Review Board of the Harley Street Children's Hospital (HSCH) in London, United Kingdom. Informed consent was obtained from parents or legal guardians.

### Patient population

We searched the oncology database of HSCH for all patients with diffuse midline gliomas of the pons (DIPG), who had been treated with CED infusions of carboplatin and sodium valproate at HSCH from January 2017 to May 2020. Diffuse midline glioma of the pons was defined based on clinical and radiological criteria for inclusion into clinical trials for DIPG prior to the era of biopsy. The criteria included: enlargement of the pons with diffuse T2W signal change occupying >50% of the volume and presence of symptoms of ataxia, cranial nerve palsies or long tract signs (Sampson et al., 2007). The patient's disease within the pons was accessible for CED infusions by the neurosurgical placement of four micro-catheters avoiding eloquent anatomical structures and pathological features representing significant clinical risk such as large hemorrhage or cysts (Szychot et al., 2021).

### Renishaw drug delivery system

Patients underwent implantation of the Renishaw Drug Delivery System (RDDS) that comprised of a skull-mounted hub attached to four implantable micro-catheters. The RDDS catheters were positioned by the neurosurgeon (SG) to optimize distribution of the infusate to the pontine region and peduncles. Two catheters were implanted trans-frontally through the cerebral peduncles to cover the central portion of the pons and two trans-cerebellar catheters were implanted to cover the lateral portions of the pons as previously described (Szychot et al., 2021).

### Intermittent convection-enhanced delivery

All selected patients underwent treatment with the CED of different combinations of sodium valproate and carboplatin, including monotherapy. The unique neurological side effect profile of each catheter was established at the first treatment cycle. On day 1 of cycle one, the profile of each catheter was determined by infusing down all catheters until side effects (i.e., sixth nerve palsy, ataxia, headache) were induced. Infusions of carboplatin and sodium valproate were initiated at 0.03 mL/h and gradually increased to the maximum rate (0.24–0.3 mL/h) over an hour as previously described (Szychot et al., 2021) and the total volume of infusate delivered was recorded.

### Imaging

The availability of MRI images at diagnosis, 6–8 weeks following radiation therapy, after catheter implantation, and following day 1 of first cycle of CED infusion was mandatory for inclusion in the study.

#### Image acquisition

Contrast-enhanced MRI was performed on a 1.5-T scanner (Avanto; Siemens Healthineers, Erlangen, Germany). The imaging protocol consisted of fat suppressed 3D pre and post gadolinium T1W, 3D T2W, 3D FLAIR and diffusion-weighted imaging (DWI) with b values 0 and 1,000. ADC maps were automatically generated by the scanner. Please see Table 1 for image acquisition parameters.

**Table 1:** MRI acquisition parameters.

**Sequence**		**TR**	**TE**	**TI**	**ACQ**	**Ave**	**ACQ matrix**	**Slices**	**Slice thickness (mm)**
DWI	SE-EPI	9,700	99	-	2D	2	192 x 192	39	3
FLAIR	IR	5,000	125	1,800	3D	2	512 x 512	144	1
T2W	SE	3,000	408	-	3D	2	256 x 224	176	0.9
T1W	IR	2,000	2.42	1,100	3D	1	256 x 256	176	0.9

SE, Spin Echo; EPI, Echo-Planar Imaging; IR, Inversion Recovery; TR, Time to Repetition; TE, Time to Echo; TI, Time to inversion; Acq, Acquisition; Ave, number of averages.

### Image analysis

#### i) Qualitative

MRI studies were reviewed by a Neuroradiologist with 10 years' experience (HH) pre and post infusion. The following MRI characteristics were noted on the baseline MRI study: presence of enhancement, diffusion characteristics (facilitated, restricted), definition of tumor margin (ill-defined, well-defined), eccentricity, involvement of the midbrain, medulla and middle cerebellar peduncles. An increase or decrease in area of signal abnormality and ADC was noted on the MRI study post CED infusion.

#### ii) Quantitative

##### Volumes of interest

**Figure 1:**
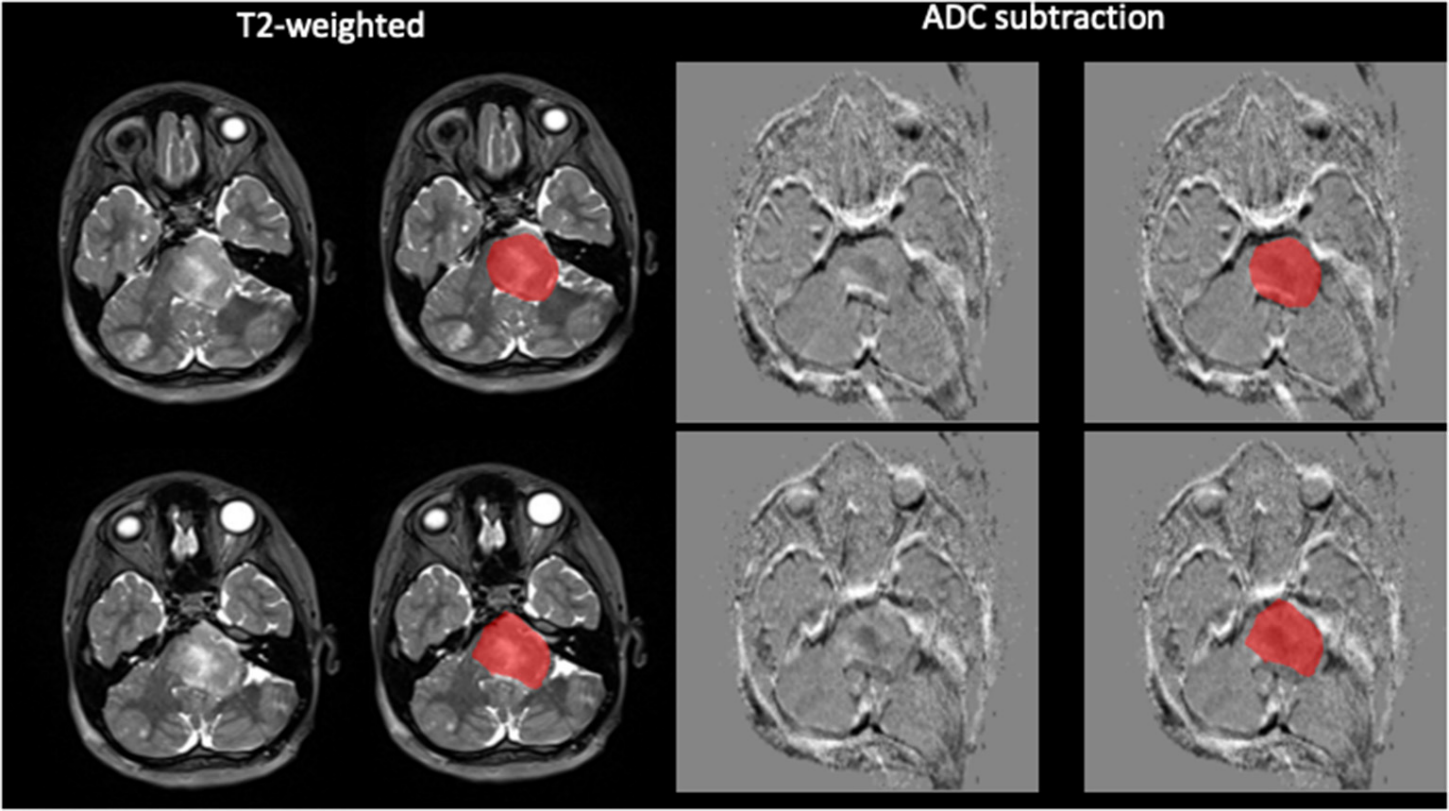
Demonstration of semi-automated tumor segmentation. Tumor segmented on T2W image using normal appearing white matter signal intensity as threshold. ADC subtraction image with the segmentation superimposed.

Lesion masks were created using the semi-automated segmentation tool in ITKsnap which is more reproducible than manual segmentation. The lesion masks were created in native space using the T2W 3D sequence (which has isotropic voxels, optimal for segmentation) and the ADC map. The following pipeline was used: (i) *Active Contour Tool* was selected to encompass the lesion and in the *Segment 3D* panel the default thresholds were adjusted to produce the *speed image*, lower threshold approximately that of normal appearing white matter and upper threshold approximately that of CSF; (ii) *Initialization mode*: bubbles at 50% opacity were placed in various regions of the lesion using the *Add Bubble at Cursor* tool; (iii) *Evolution:* the bubbles were grown to fill the lesion. Using the *Active Contour Evolution Parameters* tool, the *smoothing* was set to 0.8 and *continuous update* was used to check the segmentation. Finally, the 3D segmentation tool was used to edit the lesion mask. Voxel count, Volume (mm^3^) and Intensity Mean ± SD were extracted for each segmentation. Please see Figure 1 for examples of the lesion masks (ITKsnap) (Hargrave et al., 2008).

We assumed that the difference in signal intensity (SI) between the pre-infusion and post-infusion T2W and ADC lesion masks represents the infusate-related change in water content and that the volume of the elevated difference signal represented the volume of distribution of the infusate. To account for differences in T2W SI across different MRI studies, the ratio of mean segmentation T2W SI to normal appearing white matter was calculated (T2seg/NAWM). The following data was collected on each of the pre and post infusion MRI: (i) (T2seg/NAWM); (ii) T2 segmentation volume; (iii) mean ADC of segmentation; (iv) ADC segmentation volume.

In order to estimate the percentage of tumor coverage exposed to the drug, the difference in T2 segmentation volume pre and post infusion was expressed as the percentage of the pre infusion T2 segmentation volume.

##### ADC alignment and pixel-by-pixel subtraction

The ADC maps prior to, and post infusions were aligned by using combined transformation parameters from linear registration of T1W and ADC maps within imaging sessions and between sessions for T1W scans and applying them to post infusion ADC map. Pre infusion ADC map data was subtracted from ADC post infusion map, so that any increase in ADC, hypothesized to be equivalent to the volume of infusate distribution, appeared bright in subtraction image. Nifty-reg package was used for all registrations [https://github.com/KCL-BMEIS/niftyreg].

### Statistical analysis

Statistical analysis was performed using SPSS (IBM, USA). Differences between pre and post infusion scans for the following categories were analyzed with paired *T*-test: (i) T2seg/NAWM); (ii) T2 segmentation volume; (iii) mean ADC of segmentation; (iv) ADC segmentation volume.

Association of the infusate volume and difference in pre and post infusion were assessed with Pearson correlation coefficient: (i) T2seg/NAWM); (ii) T2 segmentation volume; (iii) mean ADC of segmentation; (iv) ADC segmentation volume.

For both these analyses *P*-value of < 0.05 was considered significant.

## Results

### Patients' characteristics

Thirteen children and young people aged from 2.8 to 18.3 years (median 6.9 years) were identified. One patient was excluded from the study in view of the corrupted imaging data. Different combinations of sodium valproate and carboplatin, including monotherapy were used and are described elsewhere (Szychot et al., 2021). CED infusion was commenced within 72 h of the Renishaw device and catheters implantation in all 13 cases. Infusate was delivered to limits of patient tolerance on day 1 of the first cycle of CED therapy (mean 4.4 +/− 0.7; range 3.8–5.7 ml).

### MRI evaluation

#### Baseline tumor characteristics

##### Qualitative

Table 2 demonstrates the tumor baseline MRI features. The majority of the tumors were non-enhancing at baseline (11/12). Nine out of twelve demonstrated facilitated diffusion (increased signal compared to normal brain parenchyma). Three out of twelve tumors demonstrated foci of restricted diffusion (decreased signal intensity on ADC map compared to normal brain parenchyma). Five tumors involved the mesencephalon, five the middle cerebellar peduncles whereas one tumor involved the medulla.

**Table 2:** Baseline tumor characteristics.

**Patient**	**Enhancement**	**Diffusion**	**Tumor margins**	**Eccentric**	**Mesencephalon**	**Medulla**	**MCP**
1	−	Facilitated	Well-defined	−	+	−	+
2	−	Facilitated	Well-defined	−	+	+	-
3	−	Facilitated	Well-defined	−	+	−	-
4	−	Facilitated	Poorly defined	−	-	−	-
5	−	Facilitated	Poorly defined	−	-	−	-
6	−	Facilitated	Well-defined	−	-	−	-
7	−	Facilitated	Well-defined	+	−	-	+
8	−	Facilitated	Well-defined	−	+	−	+
9	−	Patchy restricted	Well-defined	+	+	−	+
10	+	Foci of restricted diffusion	Poorly defined	−	-	−	-
11	−	Facilitated	Well-defined	−	-	−	-
12	−	Patchy restricted	Well-defined	−	-	−	-
13	−	Facilitated	Poorly defined	+	−	-	+

MCP, middle cerebral peduncle; eccentric, tumor involving the lateral (vs. medial) aspect of the brain parenchyma; + imaging characteristic identified; − tumor characteristic not identified.

##### Quantitative

Table 3 demonstrates the quantitative segmentation characteristics as baseline. Mean ADC was 1088.7 ± 94.25 mm^2^/s, range 965.7–1147.6 mm^2^/s and mean ADC segmentation volume 19.8 ± 4.9 ml. Mean T2seg/NAWM was 2.6 ± 0.3 and mean T2 segmentation volume 19.4 ± 5.0 ml.

**Table 3:** Pre and post infusion difference in MRI parameters.

**Pt**	**Pre-infusion**				**Post-infusion**				**Difference in segmentation volume (ml)**		**Infusate vol (ml)**
	**Vol ADC**	**Mean ADC**	**Vol T2W**	**Mean T2 seg/ NAWM**	**Vol ADC**	**Mean ADC**	**Vol T2W**	**Mean T2 seg/ NAWM**	**ADC**	**T2W**	
1	20.2	1148.3	20.1	3.09	25.2	1756.2	23.3	6.1	5.0	3.2	4.0
2	26.3	1057.0	25.8	2.50	31.2	1650.1	29.1	6.8	4.9	3.3	3.8
3	28.0	996.7	27.9	3.19	32.0	1650.0	32.1	6.1	4.0	4.2	4.2
4	16.1	1194.1	16.0	2.38	21.8	1731.9	21.7	7.1	5.7	5.7	5.7
5	17.1	1005.8	16.9	2.64	22.0	1753.3	21.5	6.3	4.9	4.6	5.0
6	19.8	1204.2	19.5	2.50	24.2	1865.4	20.9	5.5	4.4	1.4	4.2
7	15.0	965.7	13.9	2.50	18.9	1475.1	18.9	5.4	3.9	5.0	5.0
8	17.5	1245.1	17.1	2.31	23.2	1834.5	22.7	6.7	5.7	5.5	5.1
9	12.0	1006.0	11.4	2.43	16.0	1749.4	15.1	6.3	4.0	3.7	3.9
10	18.9	1050.2	18.4	2.43	22.1	1647.0	21.9	5.9	3.2	3.5	3.2
11	20.8	1147.6	20.4	2.94	25.6	1836.4	24.1	6.9	4.8	3.7	4.6
12	26.0	1047.1	25.4	2.57	30.5	1843.8	30.0	6.1	4.5	4.6	4.5
Mean (SD)	19.8 (4.9)	1088.7 (94.2)	19.4 (5.0)	2.6 (0.3)	24.4 (4.9)	1732.4 (112.5)	23.4 (4.8)	6.3 (0.5)	4.6 (0.7)	4.0 (1.2)	4.4 (0.7)

ADC, Apparent Diffusion Coefficient; SI, Signal Intensity; SD, standard deviation; ml, milliliter; vol, volume; PT, patient.

#### Changes in segmentation characteristics pre and post infusion

**Figure 2:**
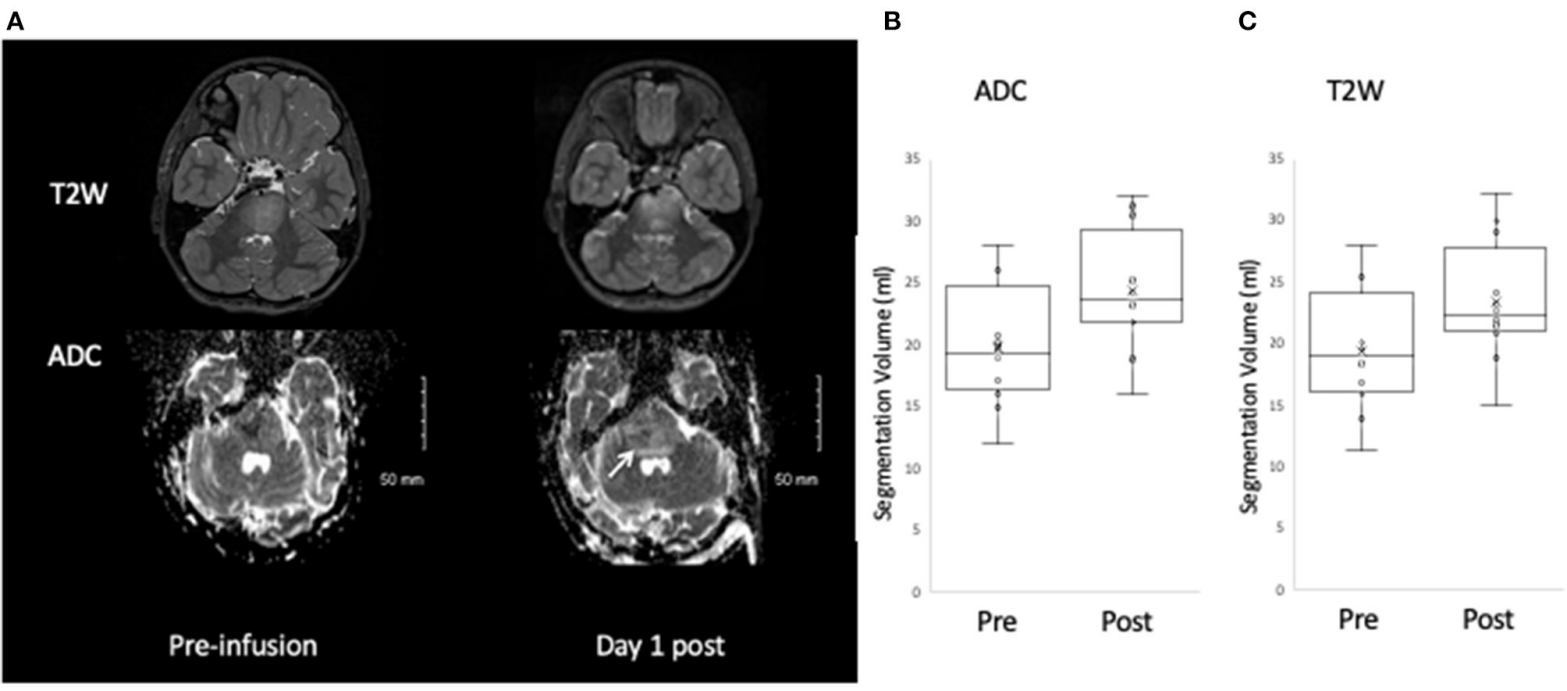
Change in T2W and ADC post infusate. An increase in the area of T2W and ADC hyperintensity is observed post infusion **(A)**, which on quantitative analysis corresponds to a significant increase in segmentation volume defined by ADC hyperintensity **(B)** and T2W hyperintensity **(C)**.

Visual inspection of the MRIs post infusion demonstrated an increased volume of T2W hyperintensity and increased volume of facilitated diffusion in all patients (Figure 2A). The VOI analysis demonstrated significant increase in T2 and ADC segmentation volumes: ADC segmentation volume pre infusion 19.8 ± 4.9 ml; post infusion 24.4 ± 4.9 ml, *p* < 0.001 (Figure 2B; Table 3); T2 segmentation volume pre infusion 19.4 ± 4.9 ml; post infusion 23.4 ± 4.8 ml, *p* < 0.001 (Figure 2C; Table 3). The SI of T2seg/NAWM increased from 2.6 ± 0.3 pre infusion to 6.3±0.5 post infusion; *p* < 0.001. Similarly, the mean ADC of segmentation increased from 1088.7 ± 94.25 mm^2^/s to 1732.4 ± 112.5 mm^2^/s; *p* < 0.001 (Figure 2; Table 3).

The average percentage of tumor exposed to drug (difference in T2 segmentation volume/pre infusion T2 segmentation volume) was 22.5% +/−9.8 range 7.1–36.0%.

#### Association between infusate volume and change in T2W/ADC segmentation parameters post infusion

**Figure 3:**
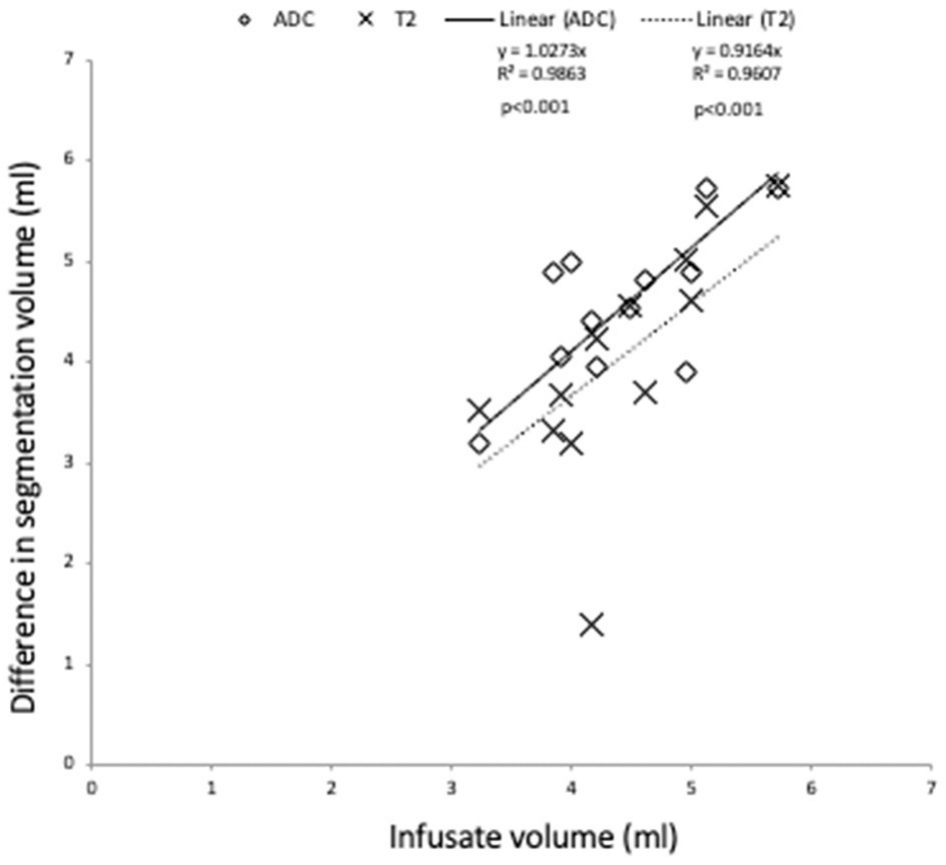
Association of change in segmentation volume pre and post infusate with infused volume. A significant correlation is observed for the infused volume and the difference in segmentation volume defined by ADC and T2W (*p* < 0.0001).

A significant correlation (*p* < 0.001) was observed for the difference in ADC and T2 segmentation volumes and the actual volume of infusate (ADC, r = 0.76, T2W, r = 0.70) (Figure 3). However, there was no significant correlation between the increase in mean ADC of segmentation or increase in SI of T2seg/NAWMW post infusion and infusate volume (ADC, r = −0.19, T2W SI ratio r = 0.41).

#### Image subtraction pre and post infusion

**Figure 4:**
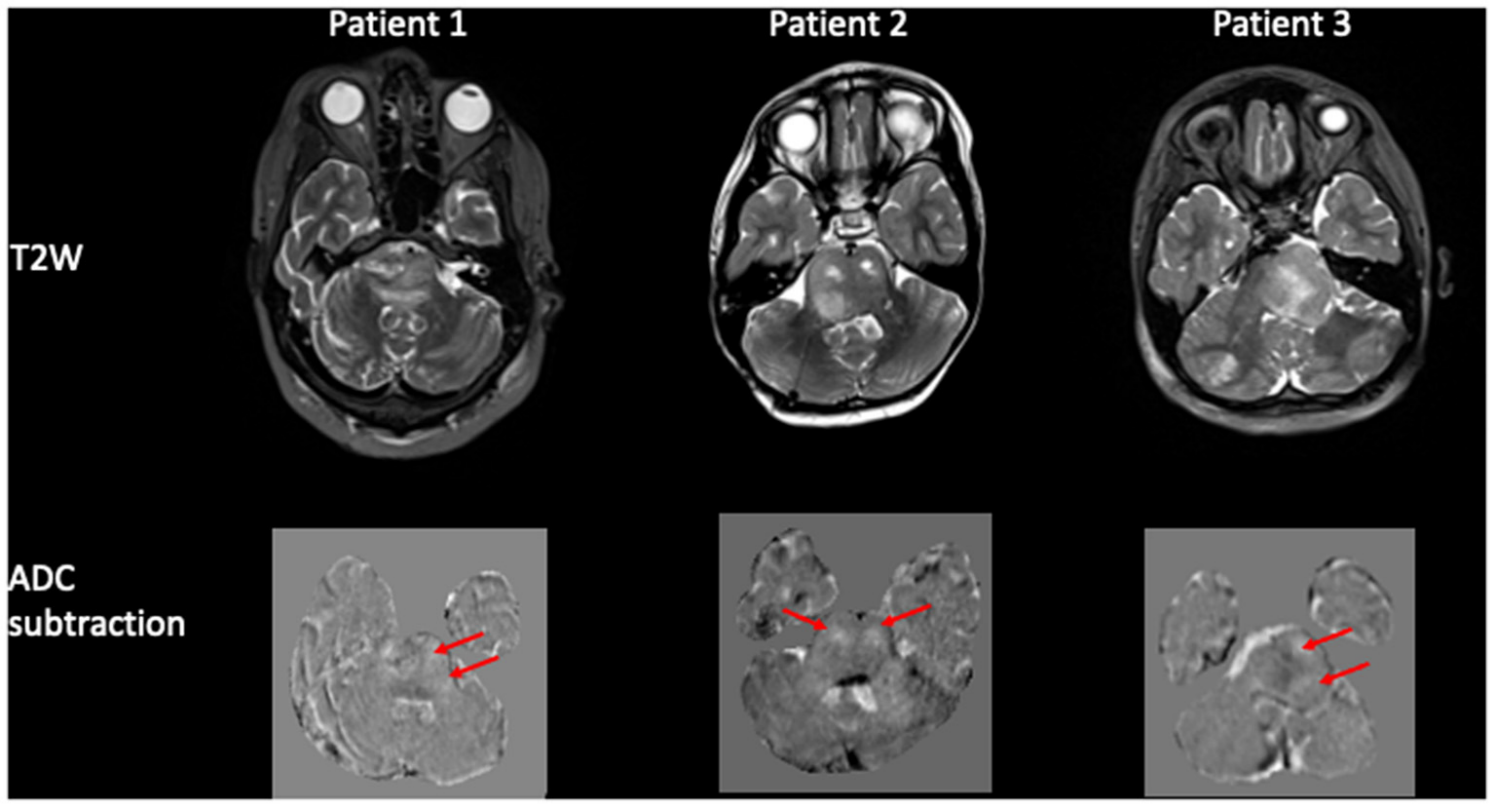
Pixel-by-pixel subtraction of ADC pre and post infusion. Panel of 3 patients demonstrating the tumor pre infusion on the T2W image and corresponding pixel-by-pixel subtraction of ADC map pre and post infusion. The difference in ADC is shown as hyperintense voxels (arrowed) within the region of the tumor.

The pixel-by-pixel subtraction of the ADC maps pre and post infusion demonstrated a region of high signal intensity encompassing the pons corresponding to the spatial distribution of the pixels with increased ADC post-infusion (Figure 4).

## Discussion

This is the first study to apply volumetric T2-weighted (T2W) and ADC to quantify infusate volume and anatomical distribution of infusate delivered by intermittent CED infusions of drugs to children and young adults with DIPG. We have shown that the difference in signal intensity between the pre infusion and post-infusion T2W and ADC images within the brainstem increases within 24 h of infusion and the difference in volume of T2W and ADC hyperintensity correlates with infusion volume. We have demonstrated that a pixel-by-pixel subtraction of ADC pre and post infusate depicts the spatial distribution of voxels with increased ADC. Our findings suggest that ADC is a surrogate marker for infusate-related changes in water diffusion and can be used in future trials to define the anatomical distribution of infusate.

The CED infusion technique seeks to maximize tumor coverage with the therapeutic infusate (Sampson et al., 2010; Phillips et al., 2012, 2014; Tosi and Souweidane, 2020). In this study patients received infusate on day 1 of the first cycle of CED therapy (mean 4.4+/− 0.7; range 3.8–5.7 ml), up to 18 ml of infusate per cycle. In order to evaluate the efficacy of the treatment, it is important to determine that the drug is being delivered to the tumor. Previously, T2-weighted imaging sequences have been used to track and approximate the volume of drug distribution (Sampson et al., 2007; Richardson et al., 2011). Similarly, Tosi and Souweidane (2020) used a change in T2W signal to estimate volume of distribution of CED infusate in a Phase 1 Clinical Trial. Our study showed that changes in ADC correlated with CED infusion volumes and subtraction of ADC maps pre and post infusion provided visual maps of the volume of infusate distribution. It is notable that the difference in ADC segmentation volumes pre and post CED infusion showed a stronger correlation with the actual volumes of infusate compared to the difference in T2 segmentation volumes (Figure 3).

Diffusion weighted imaging is an MRI method that is sensitive to the random motion of water protons (Bammer et al., 1999; Tisnado et al., 2016; Yurtsever et al., 2021). Apparent diffusion coefficient (ADC) is one of several parameters calculated from diffusion imaging and represents a measure of the average diffusion in each voxel (Maier et al., 2010). In the context of DMG of the pons in children and young adults, ADC has been used to distinguish different tumor types, predict patient outcomes and has been shown to increase following radiotherapy (Bull et al., 2012; Gutierrez et al., 2014; Ceschin et al., 2019; Roberts et al., 2020). We propose that the increased ADC signal intensity observed post infusion represents volume of the infusate distribution in the interstitial fluid within the voxel. It is possible that the increase in ADC is due to increased water diffusion in the extracellular space secondary to treatment-related cellular apoptosis rather than the infused volume. However, it is unlikely that these cellular changes would lead to a detectable increased ADC so early into treatment. Whilst an increased ADC is observed in glial tumors treated with chemo-radiotherapy, significant differences with the untreated controls were only observed on day 6 post treatment, with no significant difference on day 3 in a glioma mouse model treated with Temozolamide (Lewis et al., 2018). It is also unlikely that a correlation between the volume of infusate and change in ADC would have been observed, given the volume of extracellular water due to apoptosis would be much lower than the infusate volume of distribution.

There are several limitations of this study. As we only selected patients in whom paired, reasonably good quality imaging studies were available, our sample size was small and did not include imaging following subsequent CED infusion cycles. Secondly, we were unable to perform subtraction on the T2W images due to lack of standardization of signal intensity across MRIs. Finally, ADC volumes would not provide real-time tracking accuracy of the infusate due to large voxel size and significant differences in image distortions between two sessions in DWI data. Although we performed linear registration, the geometric distortion in EPI sequences is non-linear. We attempted to quantify this by correlating T2 infusate volume in T2 (no distortion assumed) with ADC infusate volume, resulting in a correlation coefficient of 0.39, p 0.06 demonstrating that the ADC maps are distorted. Future work will include a field map to allow non-geometric distortion correction.

Using imaging, we estimated that the mean percentage tumor coverage was 22.5% after day 1 of 1 cycle and that the ratio of infusate volume to change in T2/ADC volume is 1:1. Although the percentage coverage appears low, patients went on to have numerous further infusions to increase the coverage over several days according to our previously published protocol. Whilst we have an estimate of the percentage tumor covered by infusate, this does not necessarily correlate with percentage of drug distribution as drug pharmacodynamics have not been accounted for. Preclinical work published by Lewis et al. (2018) showed that the Vd/Vi ratio was 3.16:1 Therefore the percentage of tumor exposed to the drug may actually be nearer 75–80%. Whilst it is possible to radiolabel chemotherapeutic agents to evaluate drug delivery, on a practical level, an MRI marker would be most helpful. Although gadolinium has been used safely with a single infusion, intermittent frequent infusions every 3–8 weeks are necessary with CED, raising concern for parenchymal accumulation and renal toxicity. Given the lack of an accurate, non-invasive monitoring tool or surrogate imaging agent that could be safely co-infused, we plan to leverage recent developments in advanced imaging by focusing on evaluation of T2 mapping and multi-b value diffusion sequences which would allow quantification of water motion in different cellular compartments to improve imaging of infusate delivery (Calmon et al., 2021).

## Conclusions

This study provides the initial evidence that the voxel-by-voxel measurement of change in ADC change shows promise for illustration of anatomical distribution of infusate delivery in CED. This MRI based technique will be valuable in future clinical trials of CED infusions within brain tissue where precise anatomical localization of infusate distribution will be invaluable for demonstrating treatment efficacy.

## Data availability statement

The original contributions presented in the study are included in the article/supplementary material, further inquiries can be directed to the corresponding author.

## Ethics statement

This is a retrospective study of imaging data collected as part of a pilot study conducted under the Medicines and Healthcare products Regulatory Agency (MHRA) supervision with ethical approval of the Institutional Review Board of the Harley Street Children's Hospital (HSCH) in London, United Kingdom. Informed consent was obtained from parents or legal guardians.

## Author contributions

ES, DB, HH, SG, and DW: substantial contributions to the conception or design of the work. ES, DB, HH, and MS: acquisition and analysis. ES and HH: interpretation of data for the work and drafting the work. SG and DW: revising manuscript critically for important intellectual content. ES, HH, DB, MS, SG, and DW: provide approval for publication of the content and agreed to be accountable for all aspects of the work in ensuring that questions related to the accuracy or integrity of any part of the work are appropriately investigated and resolved. All authors contributed to the article and approved the submitted version.
